# An optimized electrochemistry-liquid chromatography-mass spectrometry method for studying guanosine oxidation

**DOI:** 10.1002/elps.201100406

**Published:** 2012-03-27

**Authors:** Robert Erb, Sabine Plattner, Florian Pitterl, Hendrik-Jan Brouwer, Herbert Oberacher

**Affiliations:** 1 Institute of Legal Medicine, Innsbruck Medical UniversityInnsbruck, Austria; 2 Antec, NV ZoeterwoudeThe Netherlands

**Keywords:** DNA damage, DNA oxidation, Electrochemistry, Liquid chromatography, Mass spectrometry

## Abstract

Oxidative stress can disrupt the integrity of genetic material. Due to its importance in the pathogenesis of different kinds of disease, including neurodegenerative disease, cardiovascular disease and cancer, major efforts are put into the elucidation of mechanisms involved. Herein, the combination of electrochemistry/liquid chromatography/mass spectrometry (EC/LC/MS) is presented as convenient, fast and simple method to study nucleic acids oxidation. Guanosine was selected as test compound. 8-Hydroxyguanosine and (guanosine-H)_2_ were identified as primary oxidation products. Oxidation was accomplished in an electrochemical thin-layer cell integrated in the flow path of the autosampler of the chromatographic system. The reaction mixture was separated and mass analyzed by LC/MS. The use of LC was found to be particularly beneficial to resolve isobaric oxidation products. Another advantage of the setup used was the ability to decouple the electrochemical cell and the electrospray ionization source from each other eliminating any kind of cell potential interaction. Separation of EC from LC/MS, furthermore, facilitates method optimization. Experimental parameters were optimized for both techniques independently. Highest yields and best detectability of oxidation products were obtained with 10 mM ammonium formate at physiological pH delivered at a flow rate of 2.5-5 μL/min through the electrochemical cell.

## 1 Introduction

DNA damage has emerged as a major culprit in cancer and many diseases related to aging [1]. DNA can be damaged by (i) spontaneous reactions, mostly hydrolysis; (ii) products of metabolism, such as reactive oxygen species (ROS) or reactive nitrogen species; and (iii) exogenous physical and chemical agents, such as ultraviolet light, ionizing radiation, toxins, pharmaceuticals, or pollutants. Produced lesions include base and sugar damages, strand brakes, crosslinks with proteins as well as the formation of bulky adducts. The cellular response to damage involves several processes [2–5], such as DNA repair, cell cycle arrest and apoptosis, while irreversible mutations contribute to oncogenesis [6, 7].

Endogenous and exogenous sources give rise to the formation of ROS (id ·O_2_^−^, H_2_O_2_, ·OH). ROS are constantly generated during oxidative respiration in mitochondria as a consequence of ionizing radiation as well as exposure to transition metals, chemicals and pharmaceutical compounds. At low concentrations, ROS function as signaling molecules [8]. At high concentrations, ROS are toxic giving rise to DNA damage. More than 100 different nucleosides resulting from oxidative stress have been isolated and characterized, which clearly indicates the complexity and diversity of nucleic acid oxidation reactions [9–11].

Methods for measuring oxidative DNA lesions can be classified into two categories [10, 12, 13]. Indirect and direct approaches are available. A very common indirect method utilizes DNA repair glycosylases to convert an oxidized DNA base into a strand brake that could be detected by reliable methods such as the comet or alkaline elution assays. Other indirect methods involve the use of antibodies or polymerase chain reaction to detect DNA lesions. The direct approaches consist first in the isolation of DNA that is then hydrolyzed enzymatically or chemically to either the nucleotide, nucleoside or nucleobase level. The mixture of DNA constituents is resolved using several stages of extraction and chromatography. Detection and characterization of formed lesions is usually accomplished by mass spectrometry (MS). Indirect and direct approaches enable the quantification of DNA lesions. The identification and structural characterization of unknown alterations is only possible with direct methods. Despite considerable success of existing tools in studying the impact of oxidative stress on genetic material, still a need for faster and simpler methods exists.

We and others have recently introduced online electrochemistry (EC)/MS to study nucleic acids oxidation [14–16]. The method consists of two parts: (i) EC is used to mimic the impact of ROS on nucleic acid species [17–19]; (ii) MS allows the characterization of the lesions produced. EC/MS is a purely instrumental method; it represents a fast, simple and convenient tool to gain insights into the mechanisms of nucleic acids oxidation. EC/MS has particularly been applied to study oxidation of guanine-containing species. Guanine exhibits the lowest oxidation potential among nucleic acid components and is therefore the preferential target of oxidation within nucleic acids. Accordingly, 8-hydroxyguanine represents the most important biomarker to assess the impact of oxidative stress on genetic material [12]. We found strong evidence that the primary products of electrochemical oxidation are species containing either 8-hydroxyguanine or cross-linked guanines [14]. Baumann et al. also detected the hydroxylated species but did not observe the cross-linked form [16]. Mautjana et al. reported on the production of cross-linked species but found no evidence for 8-hydroxyguanine [15]. We believe that to a large extent differences in the experimental setups used were responsible for the divergent experimental results. To address this issue, we have studied the impact of solvent parameters (pH and ionic strength) as well as instrumental parameters, on conversion efficiency and detectability of oxidized guanosine species. To facilitate the detection of oxidation products, liquid chromatography (LC) was used to resolve the reaction mixture prior to MS.

## 2 Materials and methods

### 2.1 Chemicals

Acetonitrile, guanosine and water were obtained from Sigma-Aldrich (St. Louis, MO, USA). Ammonium hydroxide and formic acid were purchased from Fluka (Buchs, Switzerland). All chemicals were used in the highest quality available.

### 2.2 The EC-μLC-MS system

**Figure 1:**
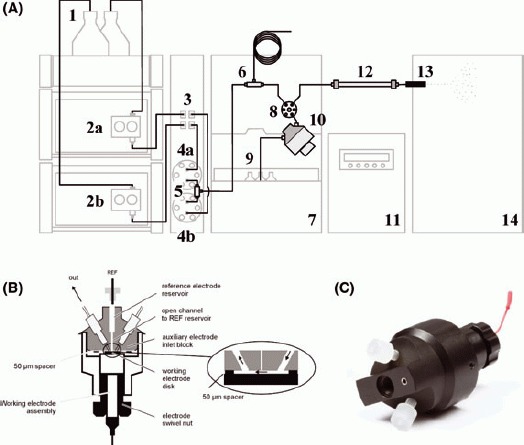
Schematic diagram of the miniaturized EC-LC-MS system and of the EC flow cell. (1) Solvent reservoirs, (2a, 2b) high pressure gradient pumps, (3) degasser unit, (4a, 4b) pressure pulsation dampers, (5) gradient mixer, (6) splitting tee-piece and restriction capillary, (7) autosampler, (8) injection valve, (9) needle, (10) EC flow cell, (11) po-tentiostat, (12) capillary column, (13) ESI source, and (14) mass spectrometer.

All experiments were performed on a modified ROXY EC/LC system (Antec, Zoeterwoude, The Netherlands) online hyphenated to a quadrupole–quadrupole–time-of-flight mass spectrometer (QSTAR XL, AB Sciex, Foster City, CA, USA). A schematic diagram of the setup is provided in Fig. 1.

Electrochemical conversions were accomplished in an electrochemical thin-layer cell (ReactorCell, Antec). The reactor cell consisted of a three-electrode arrangement including a working electrode, a counter electrode and a reference electrode. As working electrode material either conductive diamond (Magic Diamond, Antec), glassy carbon or platinum was used. The accessible area of the working electrode was 15.1 mm^2^. The inlet block of the cell was employed as counter electrode and the HyREF (Antec) electrode was used as reference electrode. The working electrode and the counter electrode inlet block were separated by a 50-μm spacer giving a cell volume of approx. 750 nL. Potentials (0–3 000 mV) were applied using a purposive potentiostat (ROXY Potentiostat, Antec).

For automated sample delivery, the reactor cell was integrated into the autosampler system [20]. The reactor cell was placed between the injection capillary and the injection valve. Sample solutions containing 20–200 μM guanosine dissolved in 0–50 mM ammonium formate (pH 2.0–8.0) were delivered through the electrochemical cell at a flow rate of 2.5 μL/min to the 2-μL sample loop. To study the impact of flow rate on conversion efficiency, a syringe pump integrated in the mass spectrometric system was used to deliver the sample solution. Flow rates between 0.5 and 15 μL/min were tested.

For all experiments a miniaturized chromatographic system was used. Chromatographic separations were accomplished on a micro column (200 × 0.2 mm id, Eurospher C18, 5 μm) prepared according to the published protocol [21–23]. A primary flow of 250 μL/min was split by a ratio of 1:100 to 2.5 μL/min with the help of a tee-piece and a 50 μm id fused-silica restriction capillary. The connection line between the gradient mixer and the splitting tee-piece was made of a 100 μm id Peek tubing; all other transfer lines had an id of 20 μm to minimize extra-column peak broadening. Chromatographic separations were accomplished with linear gradients of 2.5–30% acetonitrile in ammonium formate within 10 min. Ammonium formate concentrations varied from 1.0 to 50 mM. The pH was adjusted to 2.0–8.0 by adding different amounts of formic acid. The EC/LC system was controlled by the Clarity Chromatography software (DataApex, Prague, Czech Republic).

Eluting compounds were detected by electrospray ionization (ESI)-MS in positive ion mode, which was performed on a QSTAR XL mass spectrometer (AB Sciex) equipped with a modified TurboIonSpray source [24, 25]. The modifications included the replacements of the Peek tubing transfer line and of the stainless steel sprayer capillary by fused-silica capillaries (transfer line: 375 μm od, 20 μm id, sprayer capillary: 90 μm od, 20 μm id, Polymicro Technologies Phoenix, AZ, USA). Using a stainless-steel union, the outlet of the chromatographic column was directly connected to the transfer line of the TurboIonSpray source. Mass spectrometric parameters were optimized using a 100-μM solution of guanosine in 10 mM ammonium formate (pH 7.3) containing 50% acetonitrile (v/v). The spray voltage was set to 4.5 kV. Gas flows of 2–5 arbitrary units (nebulizer gas) and 25–30 arbitrary units (turbo gas) were employed. The temperature of the turbo gas was adjusted to 200°C. The accumulation time was set to 1.0 s. For MS/MS, the resolution of the first quadrupole was set to unit resolution. The collision gas (N_2_) flow was set to five arbitrary units. The collision energies applied depended on the fragmentation behavior of the molecule investigated. All mass spectra were acquired from 50 to 700 and recorded on a personal computer with the Analyst QS software (version 1.0, service pack 8, AB Sciex).

### 3 Results and discussion

#### 3.1 EC/LC/MS of guanosine

Online EC/MS has been applied to study the electrochemical oxidation of guanine-containing species, including guanine, guanosine and guanosine monophosphate [14–16]. The primary oxidation products seem to be species containing either 8-hydroxyguanine or cross-linked guanines of the form (guanine-H)_2_ [14]. This hypothesis is supported by results obtained in off-line experiments [17–19]. However, Baumann et al. detected the hydroxylated form only; they did not observe the cross-linked species [16]. Mautjana et al. found no evidence for 8-hydroxyguanine; they only reported the production of a dimeric form [15]. The putative dimeric oxidation product was found to be isobaric with the non-covalent dimer of guanine (=guanine_2_). Guanine is known to be self-complementary forming dimeric and tetrameric structures stabilized by hydrogen bonding [26, 27]. These complexes tend to survive the ESI process and can therefore be misinterpreted as covalently bound dimers.

To facilitate the detection and differentiation of oxidation products, EC-MS was upgraded to EC-LC-MS. There are different ways of combining EC to LC/MS. The most simple setup is the offline approach, where LC/MS is used to analyze samples manually transferred from an electrochemical reactor cell to the injection system [28–31]. Alternatively, the electrochemical cell can be integrated into the LC/MS system. Setups using the electrochemical cell either as post-column [32, 33] or as pre-column reactor were developed [34–36]. Herein, a consistent further development of the online EC/LC/MS system is presented. The electrochemical cell was integrated into the flow path of the autosampler system (Fig. 1) which enables fully automated production, separation, detection and characterization of oxidation products.

LC enables the fractionation of oxidation products prior to MS. Particularly isobaric species can be resolved. Through concentration of analytes on the column to a chromatographic peak, sensitivity is increased in comparison to direct hyphenation of EC and MS. Furthermore, chromatographic separation decreases ion suppression effects as only fractions of the reaction mixture enter the mass spectrometer simultaneously. Another advantage of the setup used is the ability to separate EC from the LC/MS part (Fig. 1). So the two electrochemical devices, the reactor cell and the ESI source, are decoupled eliminating any kind of interaction giving rise of shifts in the electrochemical potential (*E*). Separation of EC from LC/MS, furthermore, facilitates method optimization. Experimental parameters such as flow rates or solvent systems used can be optimized for both techniques independently. We selected a miniaturized chromatographic system for the separation of the oxidation products. Benefits of using such a setup include increased mass sensitivity with concentration-sensitive detectors and easier coupling with MS.

In a first set of experiments, 100 μM solutions of guanosine in 10 mM ammonium formate (pH 7.3) were electrochemically oxidized at potentials of 0–3 000 mV. The reaction mixtures were characterized by LC/MS using a gradient of 2.5–30% acetonitrile in 10 mM ammonium formate (pH 7.3) within 10 min. In all cases, subsequent LC/MS/MS experiments were used to confirm the identity of the species detected. The main oxidation products detected were 8-hydroxyguanosine and different forms of (guanosine-H)_2_. Other oxidation products previously observed in EC/MS experiments [14] were only detected in small traces. Probably, these species were unstable and decomposed within the time-frame of an LC/MS run. The difficulty to detect unstable products from analysis is a known limitation of using LC/MS to characterize reaction mixtures leaving the electrochemical cell [30].

**Figure 2:**
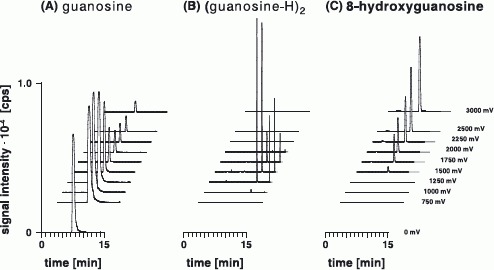
Extracted ion chromatograms of guanosine and its major oxidation products (guanosine-H)_2_ and 8-hydroxyguanosine obtained at different oxidation potentials. *E*, 0–3000 mV; flow rate through the EC cell, 2.5 μL/min; working electrode material, conductive diamond; column, Eurospher C18, 5 μm, 200 × 0.2 mm id; mobile phase (A) 10 mM ammonium formate, pH 7.3, (B) 10 mM ammonium formate containing 50% acetonitrile (v/v), pH 7.3; linear gradient, 5–60% B in 10 min; flow rate, 2.5 μL/min; scan, 50–700; sample, 100 μM guanosine dissolved in 10 mM ammonium formate, pH 7.3.

In Fig. 2 extracted ion chromatograms of guanosine, 8- hydroxyguanosine and (guanosine-H)_2_ obtained at different electrochemical potentials are shown. From these voltammograms insights into the mechanism of guanosine oxidation were gained. Guanosine oxidation started at a potential of approx. 1 000 mV. The initial oxidation of guanosine occurred in a one-electron–one-proton step to give a free radical moiety. Due to its short lifetime, the radical was not detected with our setup; it was detected indirectly. The observed formation of (guanosine-H)_2_ was a clear hint for the production of the radical intermediate. The dimeric form resulted from recombination of two radicals. As the radical had tautomeric structures, several combinations were possible to give different forms of (guanosine-H)_2_. Two isomers of the primary oxidation product were detected. The isomers were differentiated by retention times (*t_r_*(isomer 1) = 11.3 min; *t_r_*(isomer 2) = 14.3 min). These two species exhibited identical MS/MS fragmentation patterns. Cleavage of sugar moieties were the preferred fragmentation reactions. Information on the site of linkage of the two nucleobases was not retrieved from the MS/MS experiments. Maximum yields for the cross-linked species were obtained at 1250–1500 mV. At potentials beyond 1 750–2000 mV, 8-hydroxyguanosine (*t_r_* = 8.0 min) was found to be the most dominant oxidation product. This species was produced either from the primarily formed radical or from (guanosine-H)_2_ via subsequent one-electron–one-proton oxidation and addition of water.

With the EC-LC-MS setup used, we also observed guanosine_2_. This species represented a non-covalent dimer stabilized by hydrogen bonding. It was ruled out to represent an oxidation product of guanosine due to the following reasons: (i) guanosine_2_ and guanosine exhibited identical retention times, (ii) even without any electrochemical potential applied guanosine_2_ was observed, (iii) peak area ratios were constant at electrochemical potentials smaller than the oxidation potential of guanosine, and (iv) oxidation of guanosine_2_ and guanosine started at the same electrochemical potential.

#### 3.2 Optimization of the flow rate through the electrochemical cell

The flow rate of the sample solution through the electrochemical cell is a parameter influencing the conversion efficiency. Van Berkel and co-workers demonstrated that high conversion efficiencies can be obtained in EC/MS employing low flow rates [37, 38]. This is because the residence time of the analyte in the vicinity of the working electrode might be longer than the maximum diffusion time to the working electrode surface (assuming a diffusion layer thickness spanning the space between the electrodes). In our case the maximum diffusion time, *t_D_* (diffusion across the width of the cell, *d* = 50 μm), was estimated using the Einstein equation (*t* = *d^2^/2D*, where *D* is the diffusion coefficient) to be 1–2 s for nucleosides (*D* ≈ 1 × 10 ^−5^ cm^2^/s). Thus in theory, at a given cell volume of 750 nL flow rates below 15 μL/min should allow efficient oxidation of guanosine.

**Figure 3:**
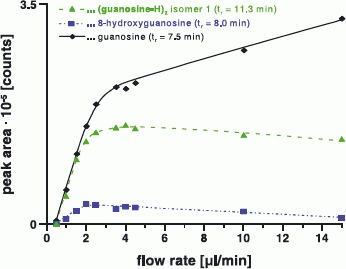
Impact of the flow rate through the EC cell on the electrochemical conversion of guanosine. E, 1750 mV; flow rate through the EC cell, 0.5-15 μL/min; mobile phase (A) 10 mM ammonium formate, pH 7.3, (B) 10 mM ammonium formate containing 50% acetonitrile (v/v), pH 7.3; linear gradient, 5–60% B in 10 min. All other conditions as in Fig. 2.

To assess the impact of flow rate on the conversion ef ficiency experimentally, guanosine oxidation was studied at flow rates ranging from 500 nL/min to 15 μL/min. The obtained results are summarized in Fig. 3. High yields of oxidation products were produced at flow rates between 1.5 μL/min and 15 μL/min. Overall best performance was obtained in the range 2.5–5 μL/min. Flow rates below 1.5 μL/min seem to be disfavored. Such low flow rates seem to enable the electrochemical decomposition of the primarily formed oxidation products.

#### 3.3 Impact of guanosine concentration on electrochemical conversion

Another parameter that might influence the conversion efficiency of the electrochemical cell is the analyte concentration. We used a thin-layer cell as reactor cell that was continuously flushed with analyte solution. Within the residence time the analyte needed to migrate to the electrode to become oxidized. Theoretically, the more analyte available, the more should be converted.

**Figure 4:**
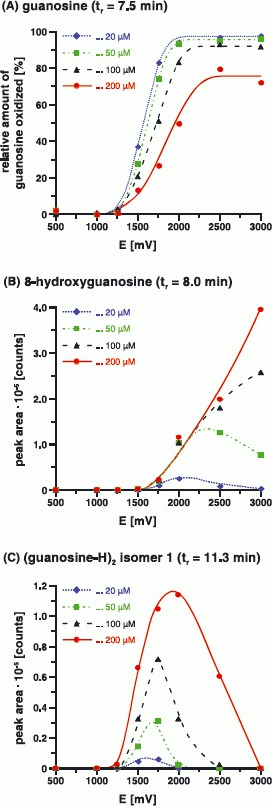
Impact of guanosine concentration on electrochemical conversion to 8-hydroxyguanosine and (guanosine-H)_2_. Mobile phase (A) 10 mM ammonium formate, pH 7.3, (B) 10 mM ammonium formate containing 50% acetonitrile (v/v), pH 7.3; linear gradient, 5–60% B in 10 min; sample, 20–200 μM guanosine dissolved in 10 mM ammonium formate, pH 7.3. All other conditions as in Fig. 2.

In Fig. 4, the effect of guanosine concentration on oxidation is shown. As expected, the higher the guanosine concentration, the higher was the amount of oxidation products produced (Fig. 4B and C). The concentration also influenced the relative amount of guanosine converted at a certain electrochemical potential. The higher the guanosine concentration, the lower was the relative amount of guanosine oxidized (Fig. 4A). The total oxidation yield decreased from 98% to 75%. This observation suggests that a maximum conversion capacity exists. The electrochemical cell can be overloaded. Loading effects can have particular implications for mechanistic studies. As shown for guanosine oxidation, overloading had a tremendous impact on mass voltammograms (Fig. 4). Half-wave potentials as well as peak potentials, which are often used as specific parameters to characterize redox systems, were shifted to higher values with increasing guanosine concentrations.

#### 3.4 Optimization of solvent and mobile phase composition

In EC/LC/MS the detectability of oxidation products can be influenced by the composition of the different solvent systems used. The conversion efficiency of the electrochemical cell depends on the properties of the sample solvent; the ESI efficiency is affected by the mobile phase composition. Particularly, pH and ionic strength of the solvents are important parameters that need to be carefully optimized to ensure a properly working EC-LC-MS system.

**Figure 5:**
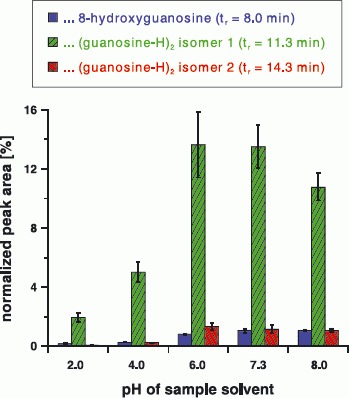
Impact of the solvent pH on the electrochemical conversion of guanosine to 8-hydroxyguanosine and (guanosine-H)_2_. *E*, 1 600 mV; mobile phase (A) 10 mM ammonium formate, pH 7.3, (B) 10 mM ammonium formate containing 50% acetonitrile (v/v), pH 7.3; linear gradient, 5–60% B in 10 min; 100 μM guanosine dissolved in 10 mM ammonium formate, pH 2.0–8.0. All other conditions as in Fig. 2.

The first parameter tuned was the pH of the sample solution. Guanosine was dissolved in 10 mM ammonium formate to reach a final concentration of 100 μM. The pH of the solution was varied between 2.0 and 8.0. Electrochemical oxidation was accomplished at 1 600 mV to ensure simultaneous conversion of guanosine to 8-hydroxyguanosine and (guanosine-H)_2_. The oxidation products were separated using a linear gradient of 2.5–30% acetonitrile in ammonium formate (pH 7.3) within 10 min and detected by ESI-MS. The peak areas of the oxidation products were determined within the extracted ion chromatograms and normalized against guanosine area. The obtained results are summarized in Fig. 5. Overall highest yields of oxidation products were obtained around the physiological pH. Acidic solvents were disfavored. A drop of the normalized peak area was observed, which suggests that extended protonation of guanosine has a negative effect on conversion efficiency.

**Figure 6:**
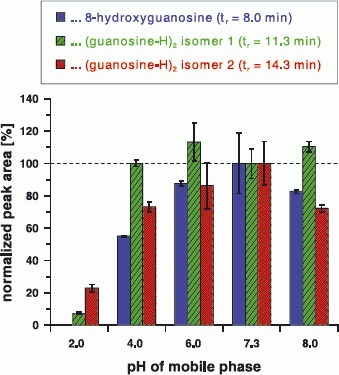
Impact of the mobile phase pH on the detectability of the major oxidation products of guanosine. *E*, 1 600 mV; mobile phase (A) 10 mM ammonium formate, pH 2.0–8.0, (B) 10 mM ammonium formate containing 50% acetonitrile (v/v), pH 2.0–8.0; linear gradient, 5–60% B in 10 min. All other conditions as in Fig. 2.

In a second set of experiments, the pH of the mobile phase was varied. 100 μM solutions of guanosine dissolved in 10 mM ammonium formate (pH 7.3) were used as samples for electrochemical oxidation. The oxidation products were separated using a linear gradient of 2.5–30% acetonitrile in ammonium formate within 10 min and detected by ESI-MS. The pH of the mobile phase was varied between 2.0 and 8.0. The peak areas obtained at pH 7.3 were used as reference points for normalization. The normalized peak areas of the oxidation products were used to assess the impact of pH on detection sensitivity. As can be deduced from Fig. 6, best performance was obtained with mobile phases close to the physiological pH. Low ionization efficiencies were obtained with the very acidic mobile phases. At pH 2.0 for instance, the peak areas of the two forms of (guanosine-H)_2_ reached only 7–23% of the original area. 8-hydroxyguanosine could not be detected at all.

**Figure 7:**
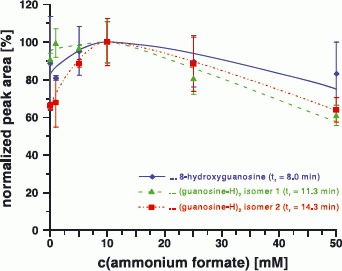
Impact of the ammonium formate concentration on the formation and detection of the major oxidation products of guanosine. *E*, 1 600 mV; mobile phase (A) 10 mM ammonium formate, pH 7.3, (B) 10 mM ammonium formate containing 50% acetonitrile (v/v), pH7.3; linear gradient, 5–30% B in 10 min; flow rate, 2.5 μL/min; scan, 50–700; sample, 100 μM guanosine dissolved in 0–50 mM ammonium formate, pH 7.3. All other conditions as in Fig. 2.

Ionic strength was another parameter whose influence on electrochemical conversion was assessed. Different amounts of ammonium formate (0.0 mM, 1.0 mM, 10 mM, 25 mM, 50 mM) were added to the sample solvent. The ammonium formate concentration of the mobile phase was kept constant at 10 mM. The impact of ionic strength on the conversion efficiency was studied by measuring the peak areas of guanosine oxidation products. In all cases, the peak areas obtained for 10 mM ammonium formate were used as reference points for normalization. The obtained results are depicted in Fig. 7. Overall best performance was gathered with 10 mM ammonium formate. All other concentrations tested led to a reduction of nucleoside species detected. The average reduction of peak area was 24% for 25 mM and 31% for 50 mM ammonium formate. At 1.0 mM ammonium for-mate the observed reduction in peak area was between 5% and 34%.

#### 3.5 Impact of electrode material on guanosine oxidation

In a final set of experiments, the impact of the electrode material on guanosine conversion was investigated. The sample was 40 μM guanosine dissolved in 10 mM ammonium formate (pH 7.3). The electrode materials tested included conductive diamond, glassy carbon as well as platinum. For each electrode material mass voltammograms were acquired. The electrochemical voltage was ramped from 0 mV to 2 000 mV. The oxidation products were separated using a linear gradient of 2.5–30% acetonitrile in ammonium formate (pH 7.3) within 10 min and detected by ESI-MS.

The experiments revealed that the electrochemical potential necessary to induce guanosine oxidation increased by approx. 500 mV in the order glassy carbon < conductive diamond < platinum. Although for all electrode materials tested a total oxidation yield of approx. 90% was reached at 2 000 mV, major differences within the amounts of oxidized species produced were observed. Highest yields of (guanosine-H)_2_ and lowest yields of 8-hydroxyguanosine were obtained with the conductive diamond electrode. The overall best conversion efficiency for guanosine to 8-hydroxyguanosine was obtained with platinum. For glassy carbon and platinum electrodes (guanosine-H)_2_ represented a minor oxidation product.

### 4 Concluding remarks

EC/LC/MS was used for studying guanosine oxidation. 8-hydroxyguanosine and (guanosine-H)_2_ were identified as primary oxidation products. A miniaturized LC system was employed for chromatographic separations. LC enabled the differentiation of otherwise undetected isomeric species. The electrochemical cell was integrated into the autosampler of the LC-MS system. Decoupling of EC and LC-MS is advantageous particularly due to the ability to optimize experimental parameters (such as flow rates and solvents) for both systems independently. Tuning of the solvent composition revealed that best performance with respect to conversion efficiency and detection sensitivity can be obtained with 10 mM ammonium formate at pH 7.3. Solvents with low pH as well as low ionic strength were found to disfavor the production and detection of oxidation products. The observed solvent effects may account for the variation of experimental results reported in terms of detectability of 8-hydroxyguanine and (guanine-H)_2_ [15, 16].

## Abbreviations

EC: electrochemistry
ROS: reactive oxygen species
